# Xanthohumol Triggers Pyroptotic in Prostate Cancer Cells via the Caspase-3/GSDME Signaling Pathway

**DOI:** 10.3390/ijms262110347

**Published:** 2025-10-24

**Authors:** Jiayin Sun, Shi Li, Zheng Duan, Hao Yu, Junjie Zhang, Jun Xue, Zhongqing Wei

**Affiliations:** The Second Clinical Medical School of Nanjing Medical University, Nanjing 210011, China; sjy98930@163.com (J.S.); ls2024@stu.njmu.edu.cn (S.L.); duanzheng0716@163.com (Z.D.); yehovah0822@163.com (H.Y.); zhangjj980721@163.com (J.Z.)

**Keywords:** GSDME, Xanthohumol, pyroptotic, prostate cancer

## Abstract

Xanthohumol (XN), a naturally occurring flavonoid characterized by the presence of prenyl moieties and obtained from hop plants (*Humulus lupulus* L.), has garnered growing interest in the scientific community owing to its diverse biological activities, including anti-inflammatory, anticancer, and antioxidant effects. However, its antitumor mechanisms, especially the inhibitory impact and related molecular pathways in prostate cancer, are not yet fully elucidated. This study investigated the effects of XN on prostate cancer and explored its underlying molecular mechanisms. The antiproliferative effect of XN on prostate cancer cells was assessed using the sulforhodamine B assay. Cellular morphological changes were examined by microscopy. Pyroptosis induction following XN treatment was evaluated via flow cytometry and Western blot analysis. Following treatment with XN, prostate cancer cells exhibited characteristic morphological changes consistent with pyroptosis. Protein analysis revealed that XN triggers pyroptosis primarily via the caspase-3/GSDME. The attenuation of XN-induced, GSDME-dependent pyroptosis by the caspase-3-specific inhibitor *Z-DEVD-fmk* further supported this mechanism. Furthermore, our results indicate that XN promotes the accumulation of reactive oxygen species (ROS) and reduces mitochondrial membrane potential, thereby activating the mitochondrial intrinsic pathway and leading to cytochrome c release, which subsequently triggers caspase-3 activation and the cleavage of GSDME, and ultimately induces pyroptosis. XN induced pyroptosis in prostate cancer cells through the mitochondrial intrinsic pathway, offering novel strategic insights for the treatment of prostate cancer and the development of innovative therapeutic agents.

## 1. Introduction

Prostate cancer ranks among the most frequently diagnosed cancers in men globally, accounting for approximately 13.5% of all male cancer cases. According to the World Health Organization (WHO), over 1.27 million new cases were reported worldwide in 2018, positioning it as the fifth most common malignancy across all cancer types, representing 7.1% of total cancer incidence [1]. The pathogenesis of prostate cancer involves a spectrum of genomic alterations, such as dysregulated oncogene signaling, functional impairment of tumor-suppressor genes, and abnormal control of apoptotic pathways. Well-established non-modifiable determinants of the disease encompass advanced age, ethnic background, familial predisposition, and inherited genetic mutations. Other significant risk factors involve conditions related to metabolic syndrome, elevated body mass index (obesity), and tobacco use [2]. Current therapeutic strategies for early-stage prostate cancer consist of radical prostatectomy, androgen deprivation therapy, and radiotherapy. In cases of advanced disease, chemotherapy serves as the mainstay of treatment. While these interventions enhance survival outcomes, they are frequently accompanied by adverse effects. Furthermore, metastatic and castration-resistant prostate cancer (CRPC) continue to pose major therapeutic challenges due to the emergence of drug resistance. Hence, developing novel agents to address these unresolved issues remains an urgent priority [3].

Many phytochemicals derived from plants have demonstrated cytotoxic effects on tumor cells, such as terpenes [4]. Accumulating evidence indicates that a variety of natural compounds exhibit the capacity to induce cell cycle arrest, trigger apoptotic death, and inhibit the growth of malignant cells [5]. Xanthohumol is a flavonoid compound naturally present in plant cells, particularly abundant in *Humulus lupulus* L. Research on xanthohumol (XN) has been conducted for over four decades, and it is widely used in cosmetics and supplements due to its antioxidant properties [6]. It also exhibits anti-tumor activity. According to Pan L. et al. [7], XN suppresses the proliferation of multiple colon cancer cell lines through the inhibition of early tumor development and interference with carcinogen metabolic pathways. In a study by Sun et al. [8], both in vivo and in vitro models of breast cancer demonstrated that XN significantly restrains tumor growth. Although the antioxidant and antitumor activities of XN are well-documented, the underlying molecular mechanisms responsible for its antitumor effects have not been fully elucidated.

Pyroptosis is a pro-inflammatory type of regulated cell death, characterized by perforation of the plasma membrane, marked cellular swelling, and consequent rupture. This lytic process triggers the substantial release of cytokines, including IL-1β and IL-18, as well as multiple chemokines, thereby eliciting a potent immunostimulatory response. The human gasdermin protein family comprises six identified members: GSDMA, GSDMB, GSDMC, GSDMD, GSDME (also termed DFNA5), and DFNB59. With the exception of DFNB59, each gasdermin possesses an N-terminal segment that confers pore-forming capability and a C-terminal region that serves an autoinhibitory function. Under normal conditions, the C-terminal domain binds to and represses the N-terminal domain, thereby preventing pore assembly and maintaining membrane stability [9]. GSDME has been identified as a key executor of pyroptosis. Upon activation, caspase-3 cleaves GSDME, liberating its N-terminal fragment (GSDME-N). This active fragment self-assembles and incorporates into the plasma membrane, leading to the formation of transmembrane pores. This permeabilization disrupts ion gradients, causing water influx, cell swelling, and rupture, collectively culminating in pyroptotic cell death [10]. Notably, pyroptosis is classified as immunogenic cell death and is increasingly explored as an innovative therapeutic strategy in oncology, owing to its dual capacity to directly kill tumor cells and stimulate potent antitumor immunity.

In this study, we demonstrated that XN induced reactive oxygen species (ROS) generation in prostate cancer cells under in vitro conditions, resulting in mitochondrial membrane potential depolarization, cytochrome c release, and ultimately pyroptosis through the caspase-3-GSDME pathway. These findings establish a foundation for further investigation of pyroptosis in oncology and offer a new theoretical basis for the development of therapeutic interventions against prostate cancer.

## 2. Results

### 2.1. XN Exhibits Anti-Proliferative Activity Against Prostate Cancer Cells

**Figure 1:**
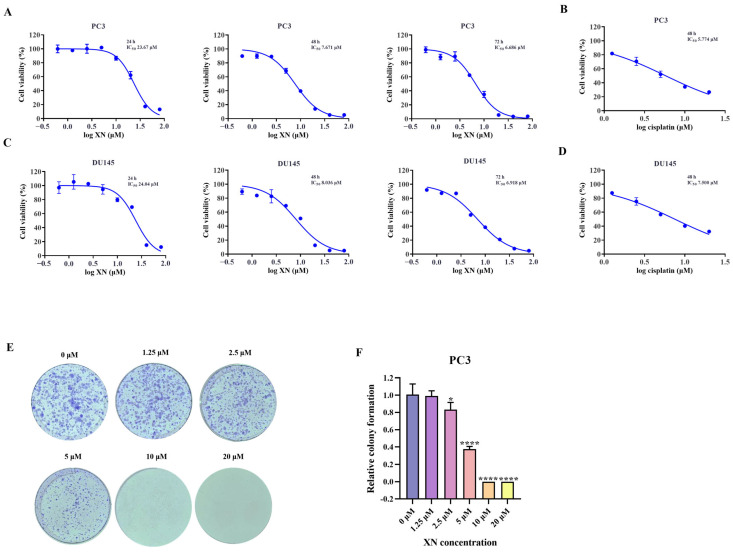
XN exhibits anti-proliferative activity against prostate cancer cells (**A**) The cytotoxicity of XN against PC3 cells. Viability of PC3 cells following 24, 48, and 72 h of XN treatment, assessed by SRB assay. (**B**) PC3 cells were exposed to cisplatin for 48 h and the cell viability was determined by SRB assay. (**C**) The cytotoxicity of XN against DU145 cells. Viability of DU145 cells following 24, 48, and 72 h of XN treatment, assessed by SRB assay. (**D**) DU145 cells were exposed to cisplatin for 48 h and the cell viability was determined by SRB assay. (**E**,**F**) Colony formation capacity of PC3 cells treated with 0, 1.25, 2.5, 5, 10 and 20 μM XN. Data are shown as mean ± SD. (* *p* < 0.05 and **** *p* < 0.0001 relative to control conditions).

As observed in the SRB assay, XN suppressed the proliferation of prostate cancer cells in a manner dependent on both concentration and duration of treatment (Figure 1A,C). The inhibition rate of prostate cancer cells was significantly higher at 48 h compared to that at 24 h, and a more pronounced suppression was observed after 72 h of treatment (Figure 1A,C). The IC50 value of XN against PC3 cells was determined to be 7.671 μM at 48 h. Guided by these findings, further investigations were carried out with XN administered at multiple concentrations over a 48 h period. To further confirm the anti-proliferative activity of XN, a colony formation assay was performed. When treated with 1.25 μM XN, no significant reduction in colony number was observed. However, at a concentration of 2.5 μM, a noticeable decrease in colony formation occurred, and almost no colonies were detected when the concentration reached 10 μM (Figure 1E,F). Taken together, these findings indicated that XN exhibits significant anti-proliferative activity against prostate cancer cells.

### 2.2. XN Triggers Pyroptosis in Prostate Cancer Cells via the GSDME Pathway

**Figure 2:**
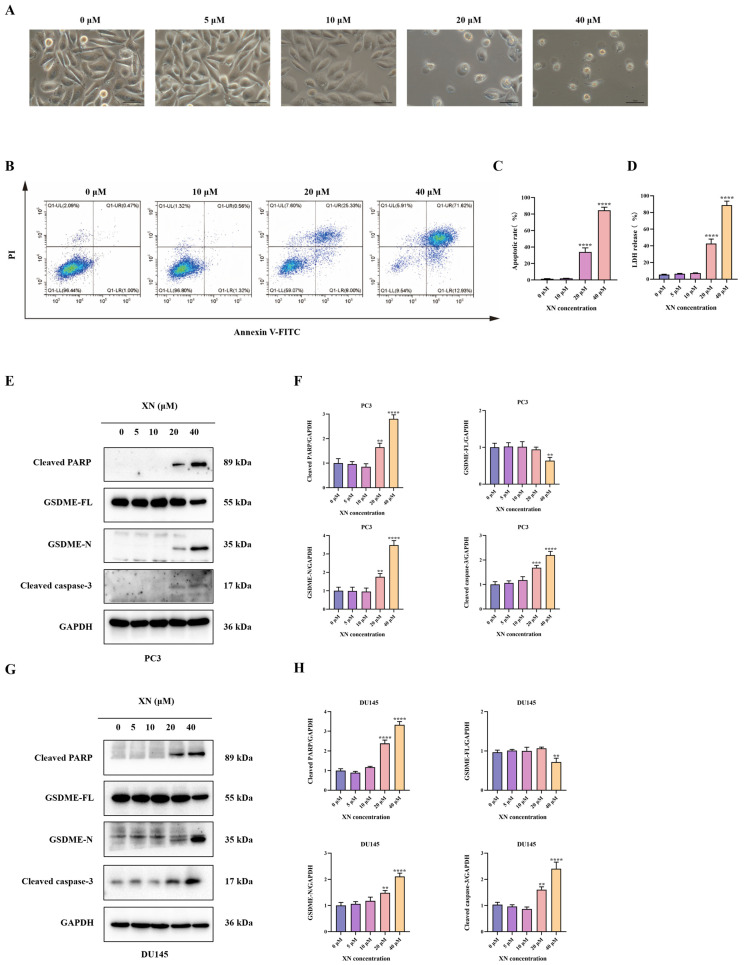
XN triggers pyroptosis in prostate cancer cells via GSDME. (**A**) Concentration-dependent morphological alterations in PC3 cells exposed to XN for 48 h (microscopic observation). (**B**,**C**) Flow cytometric evaluation of apoptosis in PC3 cells treated with XN for 48 h using Annexin V-FITC/PI double staining. (**D**) After treating PC3 cells with 0, 5 10, 20, and 40 μM XN for 48 h, measure the LDH release. (**E**,**F**) Protein expression levels of cleaved caspase-3, PARP, GSDME-FL and GSDME-N in PC3 cells following XN treatment at different concentrations. (**G**,**H**) Protein expression levels of cleaved caspase-3, PARP, GSDME-FL and GSDME-N in DU145 cells following XN treatment at different concentrations. The bar chart represents the relative protein expression levels. Data are shown as mean ± SD. (** *p* < 0.01, *** *p* < 0.001 and **** *p* < 0.0001 relative to control conditions).

Pyroptosis, a type of programmed cell death, is mainly executed by GSDM family proteins such as GSDME. In XN-treated prostate cancer cells, no significant morphological changes, such as cell swelling or membrane blebbing, were observed at low concentrations (5 and 10 μM) (Figure 2A). With increasing XN concentration (20 μM), pronounced cellular swelling and bubble-like protrusions became evident (Figure 2A). At 40 μM, massive cell swelling and membrane blebbing were observed (Figure 2A). Following drug treatment, Annexin V-FITC/PI staining showed no significant increase in double-positive cells at 10 μM XN. In contrast, a pronounced rise in Annexin V+/PI+ cells was observed at 20 and 40 μM (Figure 2B,C). The observed PI incorporation and nuclear staining in XN-treated PC3 cells point to a loss of membrane integrity, likely through the induction of pyroptotic pore formation. Disruption of plasma membrane integrity results in the release of cytoplasmic contents such as LDH, which is retained intracellularly under normal conditions. The LDH release assay indicated a significant increase after exposure to 20 and 40 μM XN, reflecting loss of membrane integrity. By comparison, treatments with 5 or 10 μM XN did not induce notable LDH release (Figure 2D). Emerging evidence indicates that some Natural compounds can induce pyroptosis in malignant cells by facilitating caspase-3-mediated cleavage of GSDME to release its active N-terminal fragment [11]. Consistent with this mechanism, Western blot analysis showed that XN, at concentrations of 20 and 40 μM, enhanced the cleavage and activation of both caspase-3 and PARP in PC3 and DU145 cells (Figure 2E–H). Moreover, XN treatment led to the accumulation of the N-terminal fragment of GSDME (N-GSDME), which is responsible for executing pyroptosis, further confirming the activation of this cell death pathway (Figure 2E–H). Taken together, these findings imply that XN promotes pyroptosis in prostate cancer cells primarily through the GSDME.

### 2.3. XN Triggers Pyroptosis in PC3 Cells via the GSDME Pathway

**Figure 3:**
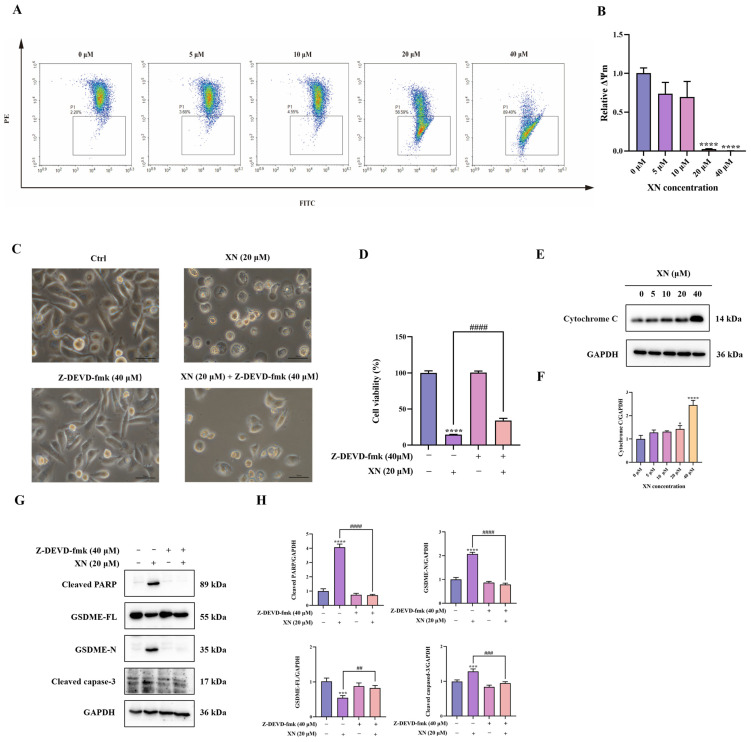
XN induces GSDME-dependent pyroptosis by activating the mitochondrial intrinsic apoptosis pathway. (**A**,**B**) PC3 cells were exposed to XN for 48 h and the MMP was determined by flow cytometry using JC-1 dyeusing. (**C**) Morphological changes in PC3 cells induced by XN for 48 h, either administered alone or in combination with *Z-DEVD-fmk*. (**D**) Cell viability of PC3 cells after treatment with XN alone or in combination with *Z-DEVD-fmk* for 48 h. (**E**,**F**) Cytochrome c expression levels was determined by Western blotting in PC3 cells under XN treatment at varying concentrations. (**G**,**H**) Expression of caspase-3, PARP, GSDME-FL and GSDME-N in PC3 cells after co-treatment with XN and *Z-DEVD-fmk*. The bar chart represents the relative protein expression levels. Data are shown as mean ± SD. (* *p* < 0.05, *** *p* < 0.001 and **** *p* < 0.0001 relative to control conditions; ## *p* < 0.01, ### *p* < 0.001 and #### *p* < 0.0001 indicate significant differences between experimental groups).

Emerging experimental support indicates that the intrinsic mitochondrial apoptotic pathway contributes significantly to the induction of GSDME-dependent pyroptosis [12]. To evaluate the participation of this pathway in XN-triggered cell death, we initially assessed alterations in MMP in PC3 cells via JC-1 fluorescence staining. Exposure to 20 μM XN provoked a conspicuous transition from red to green fluorescence, indicative of severe MMP loss (Figure 3A,B). Such MMP breakdown is established to facilitate mitochondrial outer membrane permeabilization, thereby inducing release of cytochrome c from mitochondria Correspondingly, Western blot analysis confirmed a significant increase in cytochrome c levels after treatment with 20 μM XN (Figure 3E,F). We next examined the activation of the caspase cascade by monitoring cleavage of caspase-3 and PARP. As anticipated, XN treatment resulted in a dose-dependent increase in both cleaved caspase-3 and cleaved PARP (Figure 3G,H), indicating caspase pathway activation. To further establish the functional involvement of caspase-3, we employed the specific caspase-3 inhibitor *Z-DEVD-fmk* (40 μM) in combination with XN. Co-treatment significantly suppressed GSDME cleavage (Figure 3G,H). Consistent with these molecular observations, both morphological assessment and SRB assays revealed that caspase-3 inhibition markedly alleviated XN-induced cytotoxic effects (Figure 3C,D). Collectively, these results demonstrated that XN induced GSDME-dependent pyroptosis in PC3 cells primarily through activation of the mitochondrial intrinsic apoptosis pathway.

### 2.4. XN Triggered Pyroptosis in PC3 Cells via ROS Mediated Damage

**Figure 4:**
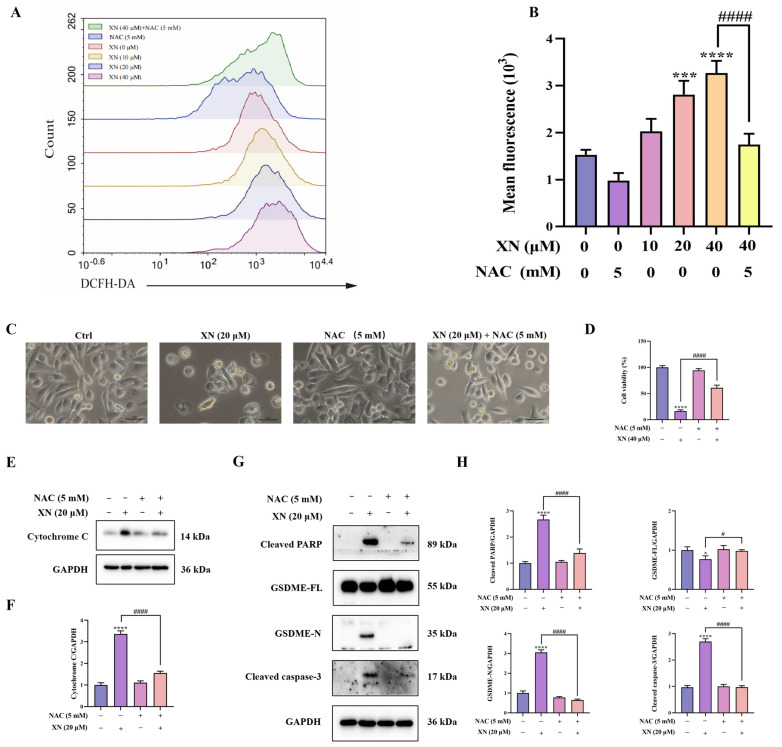
XN triggered pyroptosis in PC3 cells via ROS-mediated damage (**A**,**B**) Intracellular ROS levels in PC3 cells following 48 h treatment with XN and/or *NAC*, as assessed by flow cytometry. (**C**) Morphological appearance of PC3 cells treated with XN alone or in combination with *NAC*. (**D**) Viability of PC3 cells after treatment with XN, alone or together with *NAC*. (**E**,**F**) Cytochrome c expression levels was determined by Western blotting in PC3 cells under co-treatment with XN and *NAC*. (**G**,**H**) Protein expression of cleaved caspase-3, PARP, GSDME-FL and GSDME-N in PC3 cells following co-treatment with XN and *NAC*, analyzed by Western blotting. The bar chart represents the relative protein expression levels. Data are shown as mean ± SD. (* *p* < 0.05, *** *p* < 0.001, and **** *p* < 0.0001 relative to control conditions; # *p* < 0.05, and #### *p* < 0.0001 indicate significant differences between experimental groups).

While XN is known to trigger apoptosis through ROS-mediated mitochondrial signaling, it is not yet known whether XN-elevated ROS also contributes to pyroptosis. To investigate this, we first measured intracellular ROS accumulation using flow cytometry. The results indicated that XN treatment induced a significant increase in ROS production in PC3 cells, which was strongly suppressed by the antioxidant *NAC* (Figure 4A,B). To further investigate the contribution of ROS to XN-induced pyroptosis, PC3 cells were preincubated with *NAC* prior to XN exposure. Subsequent viability and morphological analyses showed that *NAC* pretreatment markedly attenuated XN-induced cell death (Figure 4C,D). Furthermore, Western blot analysis showed that combined treatment with XN and *NAC* reversed the release of cytochrome c, the cleavage of caspase-3 and PARP, as well as the proteolytic activation of GSDME (Figure 4E–H). These findings revealed that ROS act as a critical mediator that drives the induction of pyroptosis by XN in prostate cancer cells.

## 3. Discussion

This study showed that XN, a natural compound extracted from *Humulus lupulus* L., exerted anti-tumor effects by markedly suppressing prostate cancer cell proliferation and triggering pyroptosis in prostate cancer cells under in vitro conditions. Further mechanistic investigations indicated that the induction of pyroptosis by XN was mediated through the promotion of ROS generation and initiation of the mitochondrial intrinsic apoptotic pathway. Prostate cancer remains a serious health challenge. Although current treatments include surgical resection, radiotherapy, and hormone therapy, the disease often progresses to castration-resistant or metastatic stages, resulting in high mortality rates. There is a critical need to discover novel and more promising treatment agents and approaches [13]. Cell viability assays indicated that XN markedly suppressed the growth of PC3 cells and DU145 cells, a finding corroborated by colony formation experiments. Previous in vivo studies have demonstrated that oral administration of XN does not affect major organ function in mice and causes no adverse effects on female reproduction or offspring development in Sprague Dawley rats. These animal toxicity studies provide evidence suggesting that XN is likely to be harmless to humans [14,15]. Apoptosis has historically been recognized as a fundamental form of regulated cell death and constitutes a critical mechanism in cancer treatment strategies [16]. The anticancer effects of XN were principally linked to its capacity to impede tumor cell proliferation and elicit apoptotic cell death, an effect that had been experimentally confirmed in prostate cancer cells in prior studies [17,18,19]. In this study, we extended this conventional understanding by proposing that GSDME-dependent pyroptosis also contributed to XN’s anti-tumor effects in prostate cancer. This conclusion was substantiated by multiple lines of evidence. After 48 h of XN treatment, PC3 cells displayed characteristic morphological changes associated with pyroptosis, such as cellular swelling and bubble-like membrane protrusions. Annexin V-FITC/PI staining further demonstrated a concentration-dependent rise in the population of FITC+/PI+ double-stained cells, particularly at 20 μM and 40 μM XN. Moreover, a marked increase in LDH release was observed, confirming the loss of plasma membrane integrity. Collectively, these results demonstrated that XN suppressed prostate cancer cell proliferation and induced pyroptotic cell death.

The concept of pyroptosis was first observed in 1992 when researchers noted death in Shigella flexneri-infected mouse macrophages [20]. Subsequent studies revealed caspase-1 activation in Salmonella-induced macrophage death, which was initially misclassified as caspase-dependent apoptosis [21]. It was not until 2001 that Cookson et al. [22] distinguished this process from apoptosis: apoptotic cells maintain membrane integrity and undergo shrinkage, whereas infected macrophages exhibited membrane rupture and cell swelling. The concept of “pyroptosis” was subsequently introduced to describe this lytic and highly inflammatory mode of programmed cell death, characterized by perforation of the plasma membrane, loss of osmotic equilibrium, cellular swelling, and the extensive release of pro-inflammatory mediators [23]. A pivotal study published in 2015 revealed that pyroptosis is a programmed form of cellular demise mediated by the GSDM family of proteins [24]. Among the GSDM family, GSDMD and GSDME are the most well-characterized members. Of particular significance, caspase-3-an apoptosis-related protease-can cleave GSDME, leading to the induction of pyroptosis [25,26,27]. In this study, we examined the influence of XN on GSDME expression in PC3 cells and DU145 cells. Exposure to 20 μM and 40 μM XN for 48 h resulted in significant cleavage of GSDME. Western blot analysis showed that XN activated both caspase-3 and PARP, leading to GSDME processing. Co-treatment with *Z-DEVD-fmk* (40 μM) increased cell survival (as measured by SRB assay), reduced GSDME cleavage, attenuated cell swelling and bubbling, and decreased LDH release. These findings indicated that mitochondrial-mediated caspase activation was essential for XN-induced pyroptosis in PC3 cells. Overall, these findings indicated that XN induced pyroptosis through both caspase-3-GSDME signaling pathways. It is well documented that GSDME-dependent pyroptosis can be activated via the mitochondrial intrinsic apoptotic route. As a representative example, metformin has been shown to initiate this pathway by promoting caspase-3 activation and subsequent GSDME-N fragment generation, ultimately leading to pyroptosis in breast and liver cancer cells [28]. Similarly, alantolactone induces caspase-9 and caspase-3 cleavage via the mitochondrial intrinsic pathway, inhibiting proliferation and inducing pyroptosis in thyroid cancer cells [29]. In line with these studies, we assessed MMP in PC3 cells following XN treatment and observed a significant decrease at 20 μM XN. Collectively, these findings demonstrated that XN triggered GSDME-mediated pyroptosis in prostate cancer cells through initiation of the mitochondrial intrinsic apoptosis pathway.

**Figure 5:**
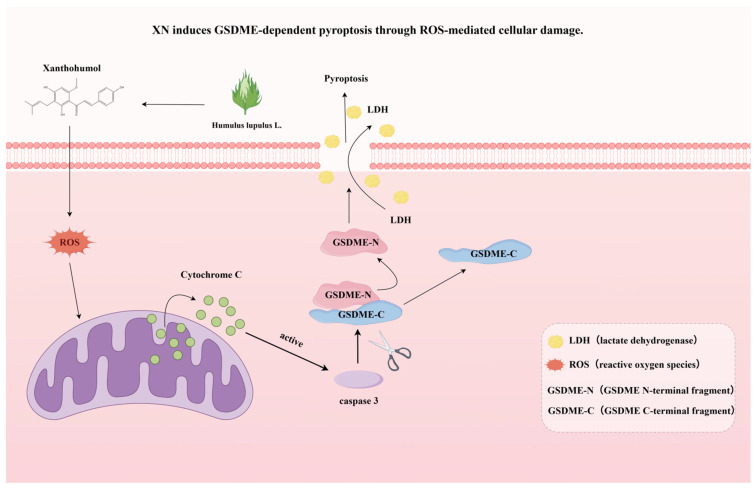
A schematic summary of this study, showing that XN induces GSDME-dependent pyroptosis through ROS-mediated cellular damage.

ROS exhibit a dual character in the context of cancer. At low-to-moderate levels, ROS contribute to tumor progression by modulating signaling cascades involved in oncogenesis and tumor suppression, inducing genomic instability, enhancing proliferative signaling, remodeling the tumor immune microenvironment, and fostering angiogenesis. In contrast, when ROS accumulation exceeds cellular antioxidant capacity, it causes extensive oxidative stress, which can activate multiple forms of regulated cell death, including apoptosis, necroptosis, and ferroptosis [30]. ROS also serve as important signaling molecules, and their overproduction has been linked to pyroptosis in various diseases [31]. For example, elevated ROS levels in cervical cancer induce NLRP3 inflammasome assembly and caspase-1 activation, ultimately leading to pyroptosis [32]. Similarly, Coxsackievirus B3 (CVB3) induces cleavage of GSDME through caspase-3 activation. Furthermore, ROS facilitate CVB3-triggered pyroptosis, demonstrating antitumor effects in colon cancer cell lines [33]. Based on these findings, we hypothesized that XN elevated intracellular ROS to induce pyroptosis in prostate cancer cells (Figure 5). Flow cytometry confirmed that XN induced substantial ROS overproduction in PC3 cells. Importantly, the ROS scavenger *NAC* reversed XN-induced pyroptosis: it attenuated the suppression of cell proliferation, reduced the cleavage of caspase-3, PARP, and GSDME, diminished cell swelling and bubbling, and decreased LDH release. Therefore, ROS likely served as a key mediator of XN-induced pyroptosis in prostate cancer cells.

## 4. Materials and Methods

### 4.1. Chemicals, Reagents and Antibodies

Commercial reagents used in this study included XN (purity ≥ 96%; Sigma-Aldrich, Taufirchen, Germany), cisplatin (Sigma-Aldrich, Taufirchen, Germany), and both *N-acetylcysteine* (*NAC*) and *Z-DEVD-fmk* (MedChemExpress, Monmouth Junction, NJ, USA). For Western blot detection, cleaved PARP, cleaved caspase-3, and GAPDH primary antibodies were procured from Cell Signaling Technology (Boston, MA, USA), while the anti-GSDME antibody was acquired from Abcam (Cambridge, UK). All secondary antibodies conjugated with horseradish peroxidase (HRP) were obtained from Cell Signaling Technology (Boston, MA, USA).

### 4.2. Cell Lines and Cell Culture

The human prostate cancer cell lines utilized in this investigation were procured from BDBIO (Hangzhou, China). PC3 cells were maintained in F-12K medium (WISENT, Nanjing, China), and DU145 cells were cultured in RPMI 1640 medium (WISENT, Nanjing, China). Both culture media were supplemented with 10% fetal bovine serum (FBS; WISENT, Nanjing, China), penicillin (100 U/mL) and streptomycin (100 mg/mL; Vazyme, Nanjing, China). All procedures, including cell culture and drug treatment, were conducted under light-protected conditions.

### 4.3. Cell Proliferation Assay and Colony Formation Assay

The sulforhodamine B (SRB) assay was employed to evaluate the effects of XN on cell viability. Cells were plated in 96-well plates and treated with varying concentrations of XN for durations of 24, 48, and 72 h. After treatment, cells were fixed through incubation with 10% trichloroacetic acid at 4 °C for 1 h. Then, 100 μL of SRB solution was added to each well and allowed to stain for 15 min. Finally, the bound dye was solubilized, and the absorbance was measured at 515 nm. For the colony formation assay, a predetermined density of cells (300–500 cells/well) was seeded into 6-well plates and subjected to the indicated treatments. After 7–8 days of culture, the cells were treated with a solution containing 4% paraformaldehyde and 0.1% crystal violet. Colony numbers were determined manually under a microscope and further subjected to statistical analysis.

### 4.4. Microscopy

PC3 cells were seeded in 6-well plates at a density of 3 × 10^5^ cells per well and treated with the drug. Subsequent morphological changes in pyroptotic cells were observed and imaged using a Leica Thunder Microscope System (Wetzlar, Germany).

### 4.5. Annexin V-FITC/PI Staining Assay

PC3 cells were seeded at an appropriate density in 6-well plates and cultured for 24 h to ensure adequate adhesion. Following this attachment period, the cells underwent treatment with specified compounds for 48 h. After treatment, cells were carefully collected and resuspended in 500 µL of specially formulated staining buffer containing FITC and propidium iodide (PI). Subsequent to a 20 min incubation conducted under light-protected conditions, all samples were examined using a novel flow cytometer (Becton Dickinson Biosciences, San Jose, CA, USA).

### 4.6. Lactate Dehydrogenase (LDH) Release Assay

According to the manufacturer’s guidelines, the amount of lactate dehydrogenase (LDH) released into the supernatant was measured with a commercially available LDH detection kit (Beyotime, Shanghai, China) to evaluate pyroptosis levels. Absorbance was read at 450 nm using a novel microplate reader.

### 4.7. Western Blot Analysis

Total protein was isolated from prostate cancer cells using ice-cold RIPA lysis buffer (NCM, Suzhou, China), and protein concentration was determined with a commercially available BCA assay kit (Beyotime, Shanghai, China). Protein separation was then performed by SDS-PAGE, followed by electrophoretic transfer onto PVDF membranes (Millipore, Billerica, MA, USA) for subsequent immunoblotting. The membranes were probed with an HRP-conjugated secondary antibody for 1 h with slow shaking. Protein signals were visualized with a novel imaging system (Bio-Rad, Hercules, CA, USA).

### 4.8. Measurement of Mitochondrial Membrane Potential (MMP)

Changes in mitochondrial membrane potential (MMP) in PC3 cells were determined with the fluorescent probe JC-1 (Beyotime Institute of Biotechnology). Cells were grown in 6-well plates and exposed to a concentration gradient of XN (0–40 μM) for 48 h. After treatment, the cells were incubated in the dark at 37 °C for the manufacturer-recommended duration. Fluorescence shifts indicative of MMP were analyzed by flow cytometry (Becton Dickinson Biosciences, San Jose, CA, USA).

### 4.9. Determination of Reactive Oxygen Species (ROS) Production

PC3 cells were plated in 6-well plates and permitted to attach for 24 h. The cells were subsequently treated with XN or *NAC* for designated durations. Following treatment, the cells were gently washed and incubated with DCFH-DA (prepared in serum-free medium) for 30 min at 37 °C under light-free conditions. Intracellular ROS levels were then quantified using flow cytometry.

### 4.10. Statistical Analysis

All numerical results are reported as mean ± standard deviation (SD). Prism 9.5 software (GraphPad, La Jolla, CA, USA) was employed for all statistical inference. Each in vitro experiment was performed in three or more biologically independent replicates. Two-group comparisons were analyzed with a two-tailed Student’s *t*-test, whereas multi-group comparisons were performed using one-way ANOVA supplemented by Tukey’s post hoc test for multiple comparisons. Differences were regarded as statistically significant when the *p*-value was lower than 0.05.

## 5. Conclusions

This study demonstrated that XN suppressed prostate cancer cell proliferation by initiating ROS-dependent pyroptosis, mediated through both caspase-3-GSDME signaling pathways. These results position XN as a potential therapeutic candidate for prostate cancer treatment. Our work clarified the mechanistic basis for the anti-tumor activity of XN and offered a theoretical framework for the development of XN-based therapies targeting prostate cancer. Several limitations should be considered in this study, including the lack of animal experiments and the unexplored issues of whether XN inhibits prostate cancer cells via other forms of cell death and its precise targets for inducing pyroptosis. These open questions will guide our future work.

## Data Availability

The original contributions presented in this study are included in the article/Supplementary Materials. Further inquiries can be directed to the corresponding authors.

## Supplementary Materials

The following supporting information can be downloaded at: https://www.mdpi.com/article/10.3390/ijms262110347/s1.
