# Supplementary figures and images for: Xanthohumol Triggers Pyroptotic in Prostate Cancer Cells via the Caspase-3/GSDME Signaling Pathway
Source: Int J Mol Sci. 2025 Oct 24;26(21):10347. doi: 10.3390/ijms262110347 (PMC12610606; doi:10.3390/ijms262110347)

Figure S1

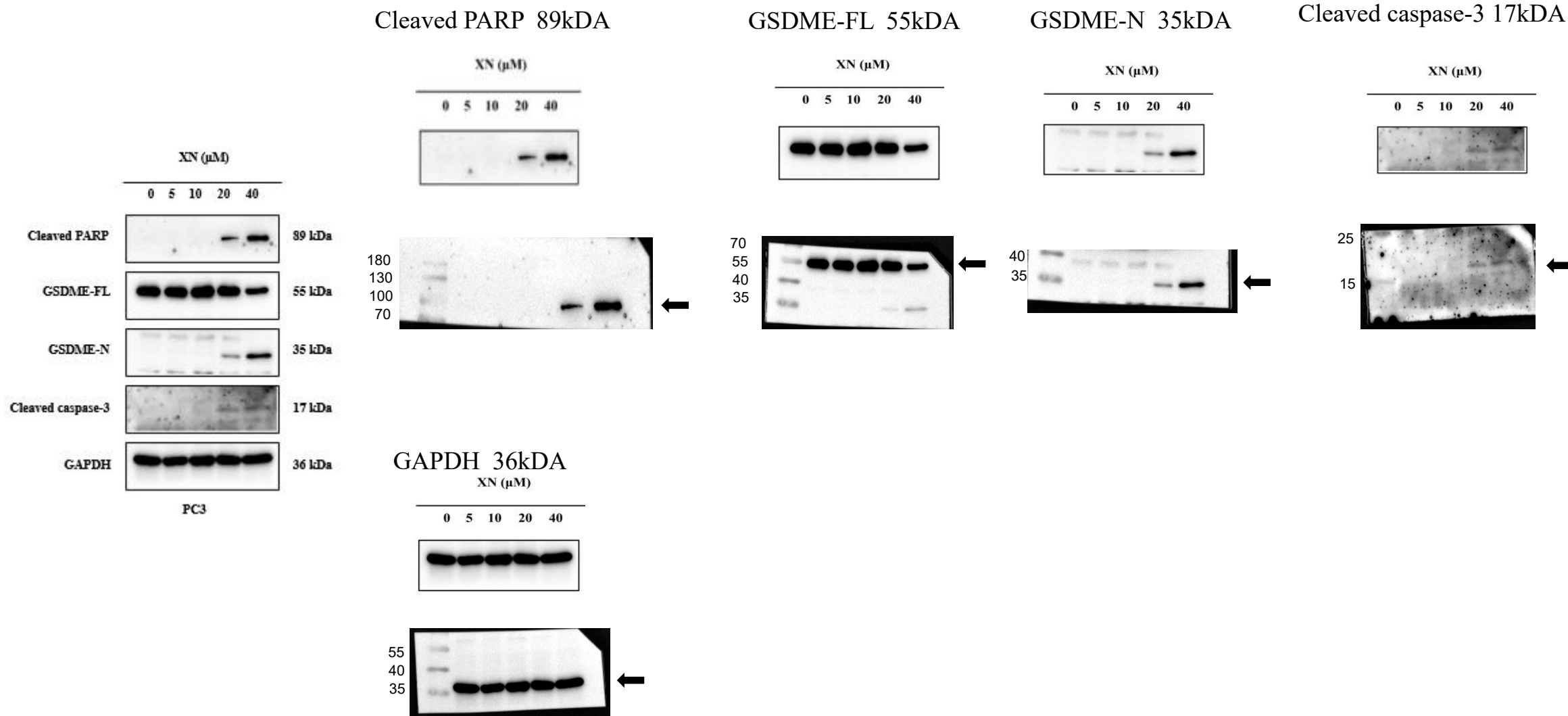

Figure S2

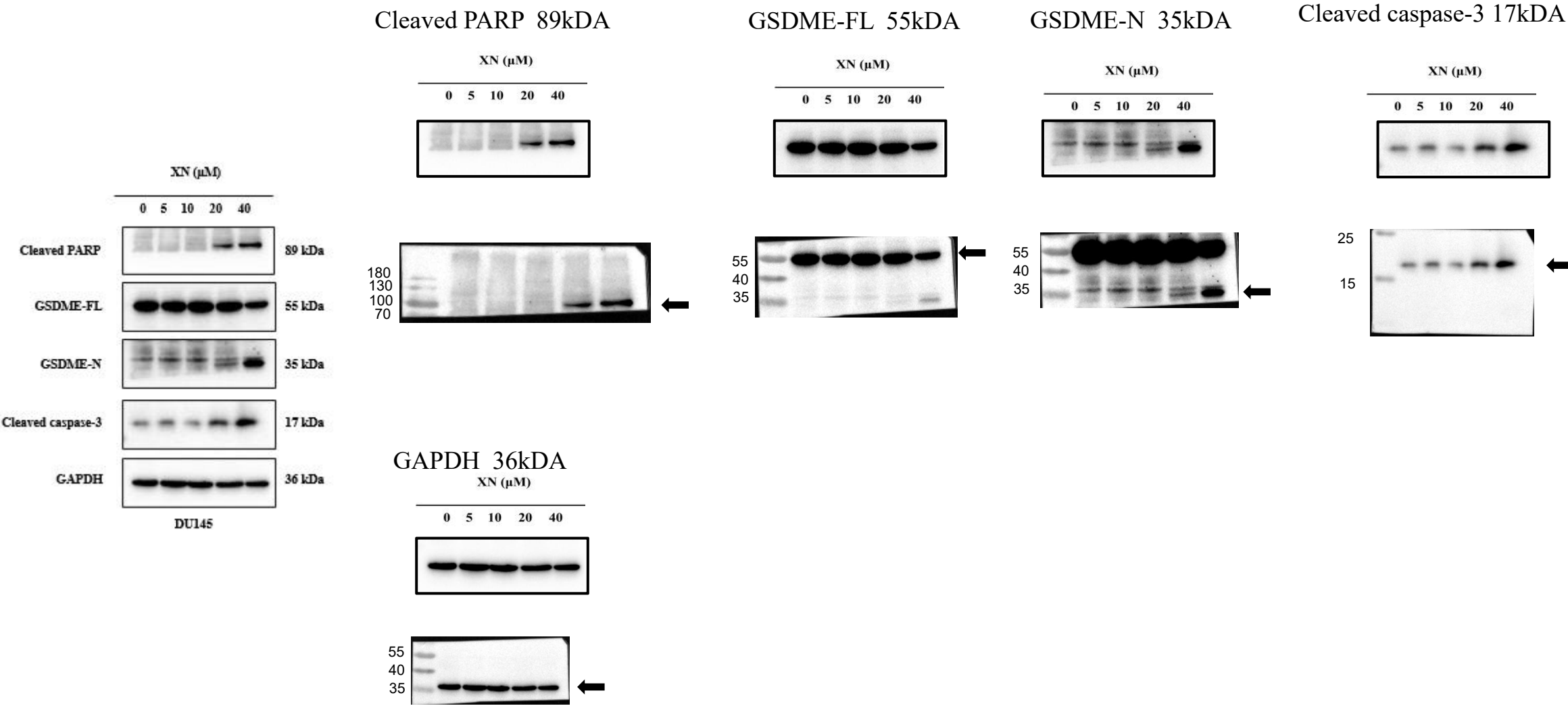

Figure S3

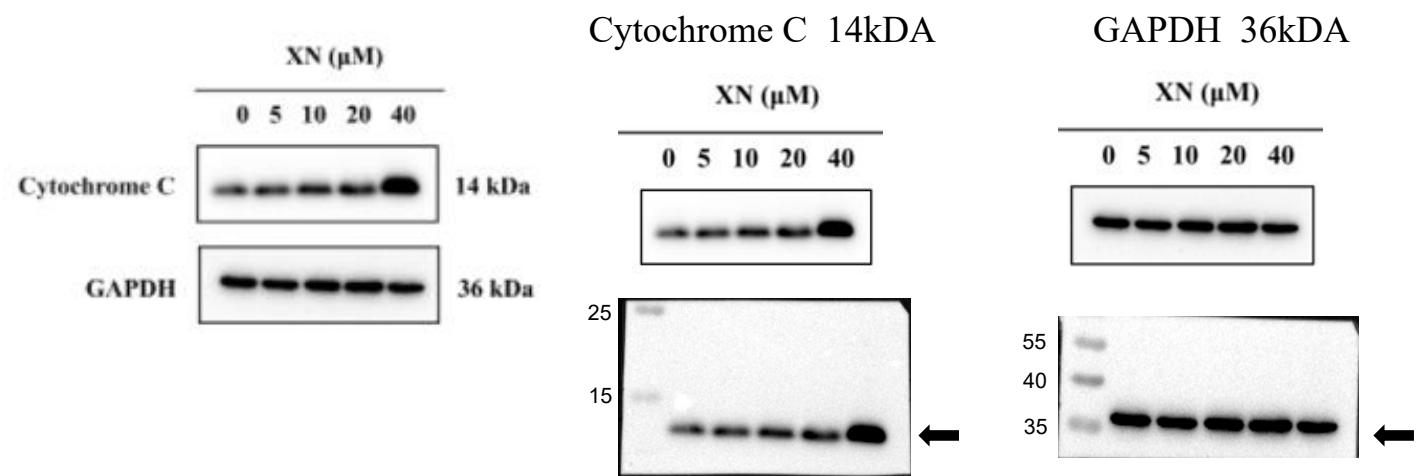

Figure S4

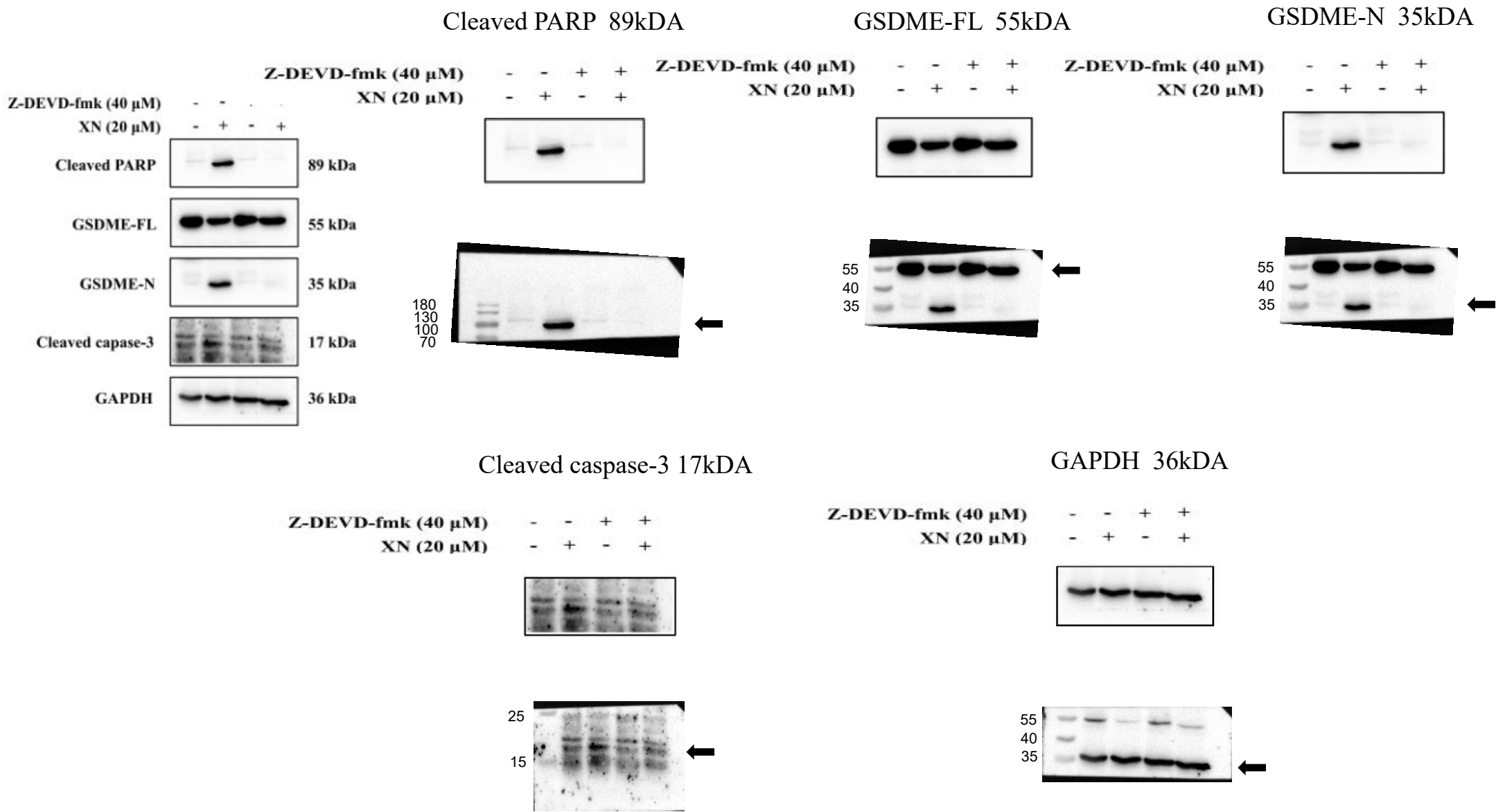

Figure S5

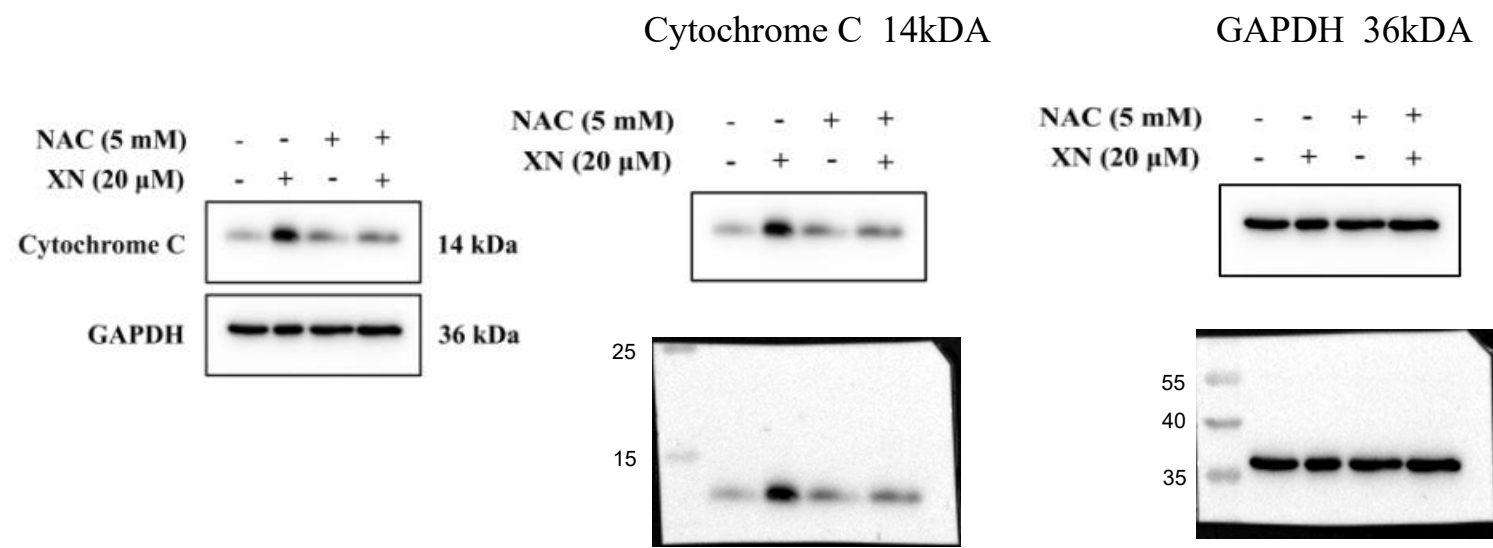

Figure S6

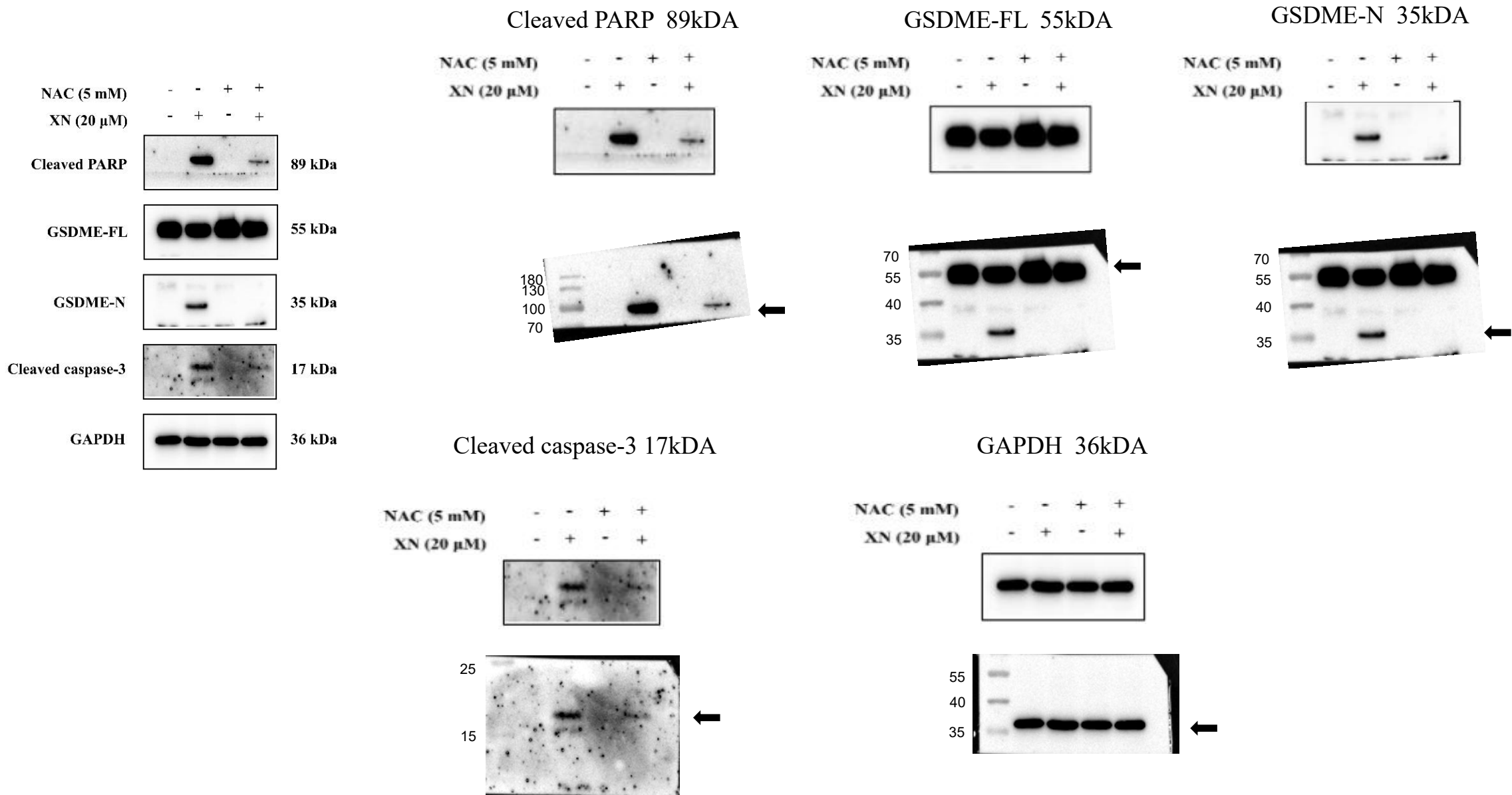

Supplement: Supplementary file 1 [file ijms-26-10347-s001.zip › ijms-3916201-supplementary.pdf]
